# p97/VCP promotes Cullin‐RING‐ubiquitin‐ligase/proteasome‐dependent degradation of IκBα and the preceding liberation of RelA from ubiquitinated IκBα

**DOI:** 10.1111/jcmm.12702

**Published:** 2015-10-14

**Authors:** Katrin Schweitzer, Alexander Pralow, Michael Naumann

**Affiliations:** ^1^ Institute of Experimental Internal MedicineMedical FacultyOtto von Guericke UniversityMagdeburgGermany

**Keywords:** NF‐κB, Cop9 signalosome, TNF

## Abstract

Cullin‐RING‐ubiquitin‐ligase (CRL)‐dependent ubiquitination of the nuclear factor kappa B (NF‐κB) inhibitor IκBα and its subsequent degradation by the proteasome usually precede NF‐κB/RelA nuclear activity. Through removal of the CRL‐activating modification of their cullin subunit with the ubiquitin (Ub)‐like modifier NEDD8, the COP9 signalosome (CSN) opposes CRL Ub‐ligase activity. While RelA phosphorylation was observed to mediate NF‐κB activation independent of Ub‐proteasome‐pathway (UPP)‐dependent turnover of IκBα in some studies, a strict requirement of the p97/VCP ATPase for both, IκBα degradation and NF‐κB activation, was reported in others. In this study, we thus aimed to reconcile the mechanism for tumour necrosis factor (TNF)‐induced NF‐κB activation. We found that inducible phosphorylation of RelA is accomplished in an IKK‐complex‐dependent manner within the NF‐κB/RelA‐IκBα‐complex contemporaneous with the phosphorylation of IκBα, and that RelA phosphorylation is not sufficient to dissociate NF‐κB/RelA from IκBα. Subsequent to CRL‐dependent IκBα ubiquitination functional p97/VCP is essentially required for efficient liberation of (phosphorylated) RelA from IκBα, preceding p97/VCP‐promoted timely and efficient degradation of IκBα as well as simultaneous NF‐κB/RelA nuclear translocation. Collectively, our data add new facets to the knowledge about maintenance of IκBα and RelA expression, likely depending on p97/VCP‐supported scheduled basal NF‐κB activity, and the mechanism of TNF‐induced NF‐κB activation.

## Introduction

An efficient, rapidly responding immune system is essential to persistently protect multicellular organisms against metabolic and environmental stresses as well as the attack by parasites and microbial pathogens. Activation of the transcription factor NF‐κB through the classical pathway of NF‐κB activation represents a centrepiece in the immediate initiation and coordination of innate and adaptive immune responses. It is triggered in response to ligation of various receptors, including cytokine receptors (*e.g*. the TNF receptor, TNFR), pattern recognition receptors (*e.g*. Toll‐like receptors, TLRs) and members of the antigen receptor family (*e.g*. the B‐cell receptor, BCR or the T‐cell receptor), by their respective ligands 1. *Via* receptor‐specific molecular pathways signals are relayed to the IKK complex, composed of two catalytic subunits (IKKα and IKKβ) and one regulatory subunit (IKKγ/NF‐κB essential modifier (NEMO)) mediating the recruitment of the IKK complex to activated receptor platforms, which acts as a common signal integrator 1, 2, 3, 4, 5. IKK complex‐catalysed phosphorylation of inhibitors of NF‐κB (IκBs), IκBα being the prototypic family member, then elicits CRL1^β‐TrCP^‐dependent ubiquitination and subsequent degradation of IκBs *via* the UPP 6, 7. During this process, NF‐κB/RelA, kept inactive in the cytosol through association with IκBs under basal conditions, becomes released, ready to enter the nucleus and activate its target genes 6, 7.

Post‐induction inactivation of NF‐κB/RelA is accomplished through various mechanisms, including NF‐κB‐induced re‐expression/re‐accumulation of IκBα in the cytoplasm 6 facilitated by the CSN 8, NF‐κB‐induced expression of the deubiquitinase (DUB) A20, contributing to upstream termination of NF‐κB activation 6, 9, and CRL2^SOCS1^ and UPP‐dependent degradation of RelA in the nucleus 10, 11, which is subject to regulation by nuclear DUBs, including the Ub‐specific peptidases (USPs) USP7 and USP48 12, 13, and the CSN 13.

The CSN is a superposed regulator of CRL assembly and catalytic activity, exerting its function by various means, including its intrinsic catalytic (NEDD8 hydrolysing/deneddylase) activity and the (reversible) association with both, CRLs and DUBs. The latter antagonize/erase Ub modifications built by CRLs on their substrate proteins 14, 15, 16, 17, 18. Reversible activating modification of CRLs with the Ub‐like modifier NEDD8 (neddylation) on a conserved C‐terminal Lys‐residue of their respective cullin (Cul) subunit (Cul1, Cul2, Cul3, Cul4A, Cul4B, Cul5, Cul7 or Parc) 19, 20 is accomplished through a three‐step enzymatic cascade reminiscent to ubiquitination 21, involving the heterodimeric NEDD8‐activating enzyme (NAE) UBA3/APPBP1 22, which is efficiently inhibited by MLN4924 23, 24, one of two NEDD8‐conjugating enzymes (UBE2M or UBE2F), and a NEDD8 ligase, ROC1 or ROC2, depending on the cullin subunit 19, 20, 22, in cooperation with a Dcn1‐like protein (hDCNL1‐hDCNL5) 22. The most efficient cullin deneddylase *in vivo* is the CSN 22.

While various molecular pathways leading to IκBα degradation and NF‐κB activation have been defined to great detail, distinct mechanistic questions remain unresolved or controversial. One of them concerns the molecular requirements for the stimulus‐induced liberation of RelA from IκBs. While UPP‐dependent degradation of IκBα is commonly viewed as a prerequisite for canonical NF‐κB activation 7, phosphorylation of RelA at Ser536 was reported in some studies to weaken the association between NF‐κB/RelA and IκBα and to mediate NF‐κB activation independent of IκBα degradation 25, 26, 27, 28. On the other hand, the molecular chaperone and segregase p97/VCP, a homohexameric member of the AAA ATPase family (ATPases associated with various activities) was recently observed to be essential for cytokine‐induced UPP‐dependent degradation of IκBα and NF‐κB activation 29. Although it is best known for its involvement in membrane traffic and fusion 30, 31 as well as ER‐associated protein degradation 32, 33, a more general requirement for functional p97/VCP in various branches of protein quality control has been observed in recent times. Mechanistically, p97/VCP and its cofactors act downstream of Ub ligases, supporting the extraction or segregation of ubiquitinated client proteins from cellular structures (*e.g*. organelles, membranes, ribosomes, chromatin or protein complexes) in an energy‐dependent fashion and co‐ordinating their processing, un‐ or refolding prior to promotion of either their liberation for subsequent reuse or their delivery to the 26S proteasome 34, 35. Particularly in case of soluble proteins however (*e.g*. IκBα 29, HIF1α 36 and the NF‐κB precursor proteins p100 and p105 37) the definite reason for p97/VCP requirement for protein turnover/degradation (or precursor protein processing in case of p100 and p105) is unknown 37, 38.

Aiming to reconcile the mechanism as well as the biochemical and molecular requirements for timely and efficient liberation of active NF‐κB/RelA from IκBα in response to TNF stimulation in epithelial cells, we thus explored in this study (*i*) the impact of RelA phosphorylation on RelA association with IκBα before and after phosphorylation and ubiquitination of the latter, (*ii*) the kinase responsible for RelA phosphorylation, and (*iii*) the requirement of p97/VCP for IκBα degradation, liberation of RelA from IκBα and RelA nuclear translocation. We observed that IKK complex‐dependent phosphorylation of RelA at serines 468 und 536 is accomplished within the NF‐κB/RelA‐IκBα‐complex simultaneous with IKK complex‐dependent phosphorylation of IκBα. Furthermore, timely and efficient liberation of RelA from IκBα essentially required UPP‐dependent degradation of IκBα. Finally, we found that timely and efficient degradation of ubiquitinated IκBα (but not IκBα‐Ub degradation at all), concomitant with timely and efficient liberation of RelA from ubiquitinated IκBα and RelA nuclear translocation, essentially depends on the presence of functional p97/VCP.

## Materials and methods

### Materials and reagents

Silencer^®^ Select siRNAs, VCP siRNA1 (s14765), VCP siRNA2 (s14767) and negative control siRNA2 (4390847), were purchased from Life Technologies (Life Technologies GmbH, Darmstadt, Germany). Other reagents were obtained from the following sources: VCP inhibitors DBeQ Calbiochem (Merck Millipore, Darmstadt, Germany), NMS‐873 MedChem Express (Hycultec GmbH, Beutelsbach, Germany), and MDBN Life Sensors (tebu‐bio GmbH, Offenbach, Germany), IKKα/β inhibitors 2‐[(aminocarbonyl)amino]‐5‐(4‐fluorophenyl)‐3‐thiophenecarboxamide (TPCA‐1) Tocris (Bio‐Techne, Wiesbaden‐Nordenstadt, Germany) and BMS‐345541 Sigma‐Aldrich (Sigma‐Aldrich Chemie GmbH, Taufkirchen, Germany), IKKε/TBK1 inhibitor MRT67307 (MedChem Express), NAE inhibitor MLN‐4924 Active Biochem (Bonn, Germany), phosphoinositide‐3‐kinase (PI3K) inhibitor wortmannin (Calbiochem), pan‐caspase inhibitor ZVAD‐fmk BD Pharmingen (BD, Heidelberg, Germany), cycloheximide (Sigma‐Aldrich) and TNF R&D Systems (Bio‐Techne, Wiesbaden‐Nordenstadt, Germany).

### Cell culture, cell treatments and siRNA transfection

Cervix carcinoma CCl2 (HeLa) cells ATCC (LGC Standards GmbH, Wesel, Germany) were cultured in RPMI 1640 medium, supplemented with FCS (10%; Biochrom GmbH (Berlin, Germany)), 2‐[4‐(2‐hydroxyethyl)piperazin‐1‐yl]ethanesulfonic acid (HEPES) (20 mM, PAA) and antibiotics (penicilline/streptomycine, 1× final concentration, PAA) in a humidified atmosphere at 37°C in the presence of 5% CO_2_. siRNA transfections were accomplished as described previously 8, using 75 nM siRNA and the SilentFect lipid reagent Bio‐Rad Laboratories GmbH (Munich, Germany). Knockdown cells were harvested the third day after siRNA transfection or at times indicated in the figures. Prior to inhibitor treatments and/or cell stimulation with TNF (10 ng/ml), as indicated in the figures, cells seeded on culture plates 1 day before were serum‐starved overnight.

### Cell proliferation assay

To analyse the impact of siRNA treatment on cell proliferation, the Cell Titer‐Glo^®^ Luminescent Cell Viability Assay Promega (Mannheim, Germany) was used according to the manufacturer's instructions. In brief: One day after targeting/non‐targeting siRNA transfection, cells were harvested by trypsination and equal cell numbers (6000/well) seeded in replicates on white, flat, clear bottom 96 well plates in a volume of 100 μl. About 4 hrs prior to analysis at day 1 to 4 after cell seeding, the culture medium was replaced with 40 μl serum‐free OptiMEM medium from Life Technologies (Life Technologies GmbH, Darmstadt, Germany) medium (Life Technologies) per well. Assays were then performed according to instructions and, after 10 min. incubation at 25°C with continuous shaking, immediately read on a Spectramax M5 plate reader Molecular Devices GmbH (Biberach an der Riss, Germany) equilibrated to 25°C and set to detect luminescence at all wavelength with an integration time of 500 msec. and a settling time of 100 msec. in an end‐point measurement. Results were blank‐subtracted against cell‐free culture medium and blank‐subtracted data processed in Excel. Applying the assay to serial 1:2 dilutions of HeLa cells, the assay was calibrated to determine cell numbers. A linear relationship between cell number and luminescence signal was obtained for up to 90,000 cells per well. Viability of siRNA‐transfected cells, seeded in parallel on 60 mm diameter petri dishes and harvested by trypsination at day 1 to 4 after seeding, was determined using Countess Cell Counter (Life Technologies). The third day post siRNA transfection RIPA lysates (see below) were prepared from harvested cells to confirm knockdown success by immunoblot (IB).

### Preparation of whole cell extracts and subcellular fractions

Procedures for the preparation of whole cell extracts (RIPA cell lysates), as well as the preparation of cytosolic, total nuclear (N_t_), soluble nuclear (N1) and insoluble nuclear fractions (N2) have been recently described in detail 13.

### Immunoprecipitation and immunoblot analysis

Immunoprecipitations (IPs) and SDS PAGE analysis were conducted as described previously 13. For IPs equal amounts of cellular protein (500–1000 μg) and 1 μg antibody per IP sample were used. Primary and secondary antibodies used in the study are listed separately (Tables S1 and S2 respectively).

## Results and discussion

### Neddylation of cullins is essential for TNF‐induced IκBα degradation and the release of RelA

Intending to reconcile the mechanism as well as biochemical and molecular requirements for the TNF‐induced dissociation of the NF‐κB/RelA‐IκBα complex in epithelial cells, the timely and efficient degradation of ubiquitinated IκBα and the concomitant liberation of active NF‐κB molecules, ready to enter the cell nucleus, we decided to initially make use of MLN4924, a potent small molecule inhibitor of the NEDD8‐activating enzyme (NAE) 24.

**Figure 1:**
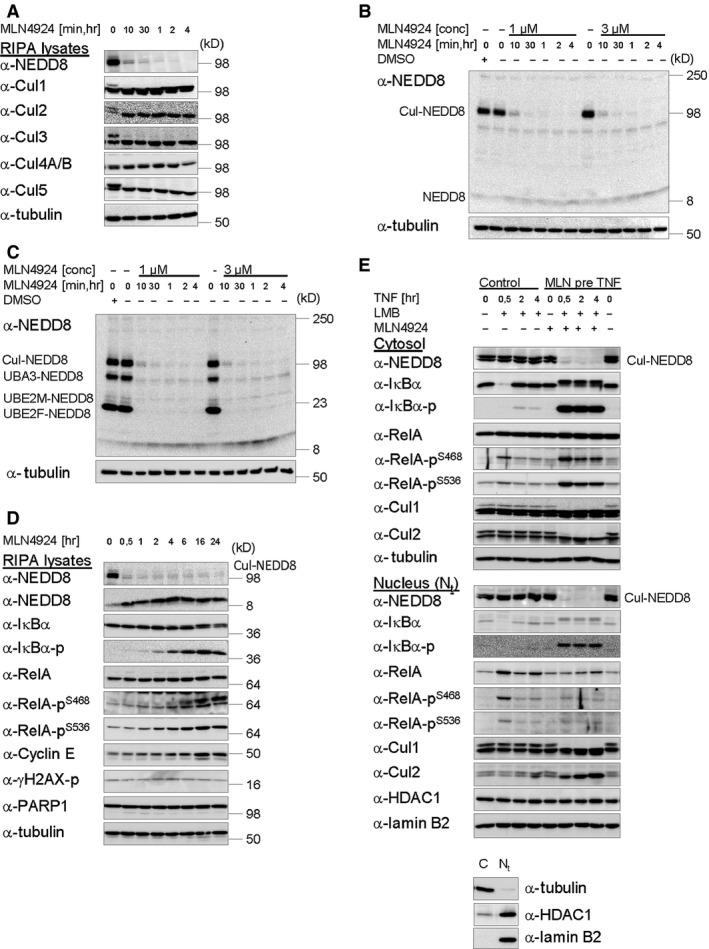
A functional neddylation pathway is essential for TNF‐induced degradation of IκBα and concomitant release and nuclear translocation of RelA. (**A**–**C**) Cullin neddylation is rapidly and efficiently inhibited by NAE inhibitor MLN4924. (**D**) Inhibition of cullin neddylation by MLN4924 in HeLa cells causes CRL substrate accumulation without induction of DNA damage or apoptosis. (**A**–**D**) After treatment with DMSO (vehicle) or MLN4924 (3 μM, or as indicated), cells were harvested by RIPA lysis at indicated times. Prior to analysis, samples were heat‐denatured by boiling in either the presence (**A**,** B**,** D**,** E**) or the absence (**C**) of β‐mercaptoethanol. (**E**) MLN4924 efficiently inhibits degradation of IκBα and RelA nuclear translocation in response to TNF. Cells treated with MLN4924 (3 μM) 10 min. prior to TNF (10 ng/ml) stimulation were harvested by subcellular fractionation at indicated times. Leptomycin B (LMB, 10 ng/ml) was added 15 min. after TNF stimulation to prevent Crm1‐dependent nuclear export of RelA. (**A**–**E**) Samples, as indicated, were analysed by IB. Detection of Tubulin (RIPA lysates and cytosol) or HDAC1 and Lamin B2 (total nuclear fractions, N_t_) was performed for control of fractionation success (**E**) and equal protein load. Lacking accumulation of (i) H2AX phosphorylated at Ser139 (γH2AX) and (ii) PARP1 cleavage upon MLN4924 treatment (**D**) indicate the absence of DNA damage and induction of apoptosis respectively.

Cullin neddylation is a highly dynamic process 24. Consistent with this notion and previous observations in HCT‐116 colon carcinoma cells 24, we observed almost complete loss of cullin neddylation within 10 min. of cell treatment with MLN4924, signified by vanishment of a band of about 98 kD, detectable with a NEDD8‐specific antibody, and verified through IB detection of individual cullins (Fig. 1A). Neddylated and non‐neddylated Cul4 failed to be properly separated, whereas particularly Cul2 appeared to strongly accumulate upon its deneddylation. Cul7 and Cul9, bearing much higher molecular weights than other cullin family members, were not analysed here, but would be expected to become deneddylated upon inhibitor treatment alike 19, 20, although NEDD8 modification of Cul7 in cells was questioned 39 and not definitely demonstrated as yet. Appropriate conditions for rapid and efficient inhibition of cullin neddylation without adverse effects on cell viability were reevaluated by subjecting cells to time course experiments in the presence of two different dosages of MLN4924 (Fig. 1B–D). Nearly, complete loss of overall cullin neddylation within 10 min. of inhibitor treatment was observed under both conditions in IBs of samples heat‐denatured in either the presence (Fig. 1B and D) or the absence (Fig. 1C) of β‐mercaptoethanol (β‐ME). The approximately 98 kD band of NEDD8‐modified cullins was the one most predominantly affected in extracts from inhibitor‐treated cells exposed to β‐ME (Fig. 1B), implicating cullins to be the primal neddylation substrates. In non‐denatured samples NEDD8‐loaded versions of UBA3 (NAE2) and the NEDD8‐conjugating enzymes (NEDD8‐E2s), UBE2M (Ubc12) and UBE2F, which cannot be separated by molecular size, were additionally detected by the NEDD8‐specific antibody and similarly affected (lost) after MLN4924 treatment (Fig. 1C) 23. MLN4924‐mediated inhibition of CRL activity through deneddylation became evident by the accumulation of CRL substrate Cyclin E 20, 40, 41, 42 as well as phosphorylated versions of IκBα (and RelA) over time (Fig. 1D), the latter notifying basal IKK activity in the absence of an exogenous stimulus 43, 44, 45. In contrast to data obtained in other human cancer cell lines derived from solid and haematologic malignancies 24, 46, 47, 48, 49, MLN4924 did not affect HeLa cell viability during the entire time course of inhibitor treatment, as determined by cell morphology (data not shown) as well as the lack of H2AX phosphorylated at Ser^139^ (γH2AX), an indicator of DNA damage 50, and cleaved PARP1 (Fig. 1D), which accumulates after caspase activation upon induction of apoptosis 51.

Having shown phosphorylated IκBα, a bonafide substrate of CRL1^β‐TrCP^ 52, 53 and phosphorylated isoforms of RelA, a substrate of CRL2^SOCS1^ 10, 11, to already accumulate upon exposure of non‐stimulated cells to MLN4924, a phenomenon previously noted in preclinical models of B‐cell‐like lymphoma 48, we next explored the impact of NAE inhibition on CRL‐dependent proteolytic turnover of components of the NF‐κB system during a time course of TNF stimulation. In cells pretreated with MLN4924, TNF‐induced degradation of IκBα in the cytosol and, to a lesser extent, in the nucleus as well, evident after 30 min. in control cells, was completely inhibited (Fig. 1E). Instead, within 30 min., IκBα was entirely converted into a more slowly migrating isoform, detectable with a phospho‐specific IκBα antibody, which thereafter persisted during the entire time course of stimulation. Simultaneously, phosphorylated isoforms of IκBε and NF‐κB precursor protein p105 (NF‐κB1) but not p100 (NF‐κB2), all being substrates of CRL1^β‐TrCP^ 54, 55, accumulated in both subcellular fractions alike (Fig. S1). Yet, in contrast to phosphorylated IκBα and p105, phosphorylated IκBε disappeared at later times (Fig. S1), likely because of dephosphorylation 56. An increase in protein expression of both precursor proteins but phosphorylation of only p105 in response to TNF is consistent with both precursor proteins being NF‐κB target genes inducible *via* the canonical pathway of NF‐κB activation 57, 58, yet only phosphorylation and processing/UPP‐dependent degradation of p105 being initiated in an IKK complex‐dependent manner 1, 59. NIK and IKKα‐dependent phosphorylation and processing of p100 in contrast is stimulated through the non‐canonical pathway of NF‐κB activation, being not actuated upon ligation of TNFR1 by its ligand TNF but upon ligation of other members of the TNFR superfamily, including lymphotoxin beta receptor (LTβR), B‐cell activating factor receptor, CD40 and receptor activator of NF‐κB by their respective ligands 1, 6.

Apart from phosphorylated IκBs, phosphorylated species of RelA accumulated in the cytosol but not the nucleus with same kinetics as phosphorylated IκBα (Fig. 1E). Although RelA phosphorylated at Ser468 noticeably decreased over time, likely because of dephosphorylation 60, this was less the case for RelA phosphorylated at Ser536. Potent MLN4924‐dependent inhibition of IκBα degradation because of inhibition of CRL1^β‐TrCP^ neddylation, and thus its catalytic activation, coincided with nearly complete inhibition of TNF‐induced RelA nuclear accumulation (Fig. 1E). Efficiency of NAE inhibition in both subcellular compartments was verified through IB detection of Cul1 and Cul2 with cullin‐specific antibodies, as well as overall cullin neddylation with a NEDD8‐specific antibody (Fig. 1E).

In summary, these data demonstrate that CRL1^β‐TrCP^ Ub ligase activity is potently and immediately inhibited upon cell treatment with MLN4924, which causes a rapid loss of CRL activating Cul1 neddylation. In consequence, in response to TNF, phosphorylated CRL1^β‐TrCP^ substrates accumulate. Representing transition states priming them for ubiquitination and subsequent proteasomal degradation under physiological conditions, they become stabilized upon deficiency in CRL1^β‐TrCP^ Ub ligase activity. Notably, the coincidence of persistent stabilization of phosphorylated IκBs (IκBα and p105) with efficient inhibition of TNF‐induced RelA nuclear accumulation already implicated at this time that RelA phosphorylation by itself is likely insufficient to liberate RelA from its cytoplasmic anchor proteins, *e.g*. IκBα. Accumulation of RelA isoforms phosphorylated at Ser468, Ser536 or both in the cytosol after treatment of cells with MLN4924, followed or not by TNF stimulation, is consistent with RelA becoming phosphorylated at these sites in the cytoplasm but de‐phosphorylated or degraded (primarily) in the nucleus, as previously suggested in some reports 10, 61, 62, 63, 64, nuclear translocation and dephosphorylation of RelA being (indirectly) inhibited by MLN4924. Published data regarding RelA phosphorylation at Ser468 are however controversial, suggesting inducible phosphorylation at this site to be accomplished/mediated by either IKKβ (the IKK complex) in the cytoplasm 64 or IKKε in the cytosol and (predominantly) the nucleus 65, 66.

### Phosphorylation‐dependent ubiquitination and proteasomal degradation of IκBα are required for its efficient liberation from associated NFκB/RelA

**Figure 2:**
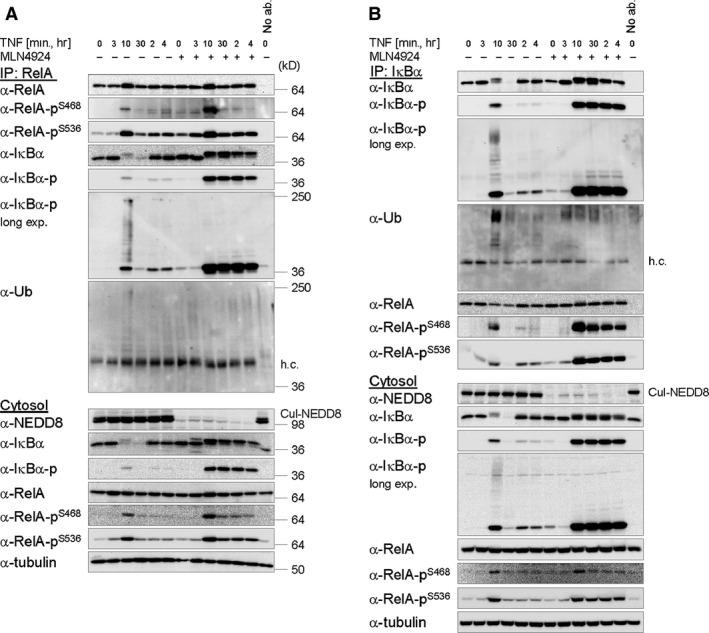
Efficient TNF‐induced dissociation of the NF‐κB/RelA‐IκBα complex requires Ub‐dependent proteasomal degradation of IκBα. (**A** and **B**) Inhibition of IκBα ubiquitination with MLN4924 stabilizes the association between (phosphorylated) IκBα and (phosphorylated) RelA after TNF stimulation. RelA (**A**) or IκBα (**B**) was immunoprecipitated from cytosolic fractions of cells treated with MLN4924 (3 μM), as indicated, 10 min. prior to TNF (10 ng/ml) stimulation. Samples, as indicated, were analysed by IB. Detection of Tubulin in the cytosol was performed for control of equal protein load.

To verify surmised physical associations between phosphorylated IκBα and RelA and its phosphorylated isoforms, reciprocal IPs of both interaction partners were performed from cytosolic fractions of cells treated with MLN4924 prior to TNF stimulation. TNF‐induced degradation of IκBα was efficiently inhibited in the cytosol, as expected. Yet, IκBα became entirely phosphorylated within 10 min. and afterwards persisted as phospho‐IκBα during the whole time course of TNF stimulation, although a slight but continuous decrease in both, phosphorylated and total IκBα, was noticed over time (Fig. 2A and B). Importantly however, phosphorylated IκBα efficiently co‐precipitated with RelA including its phosphorylated isoforms (Fig. 2A). Similarly, RelA, as well as its phosphorylated isoforms, efficiently co‐precipitated with phosphorylated IκBα, being the sole IκBα isoform in cytosolic extracts of MLN4924‐treated cells (Fig. 2B). As noted already for IκBα, phosphorylated RelA slightly but continuously decreased over time, which was most evident for RelA phosphorylated at Ser468 (Fig. 2A and B) and might be either because of dephosphorylation or residual CRL1^β‐TrCP^ Ub‐ligase activity mediating slow ubiquitination and degradation of IκBα and concomitant liberation of RelA over time, even in its deneddylated state 67. Of note however, IκBα‐Ub itself (Fig. 2B) and co‐precipitation of IκBα‐Ub with RelA (Fig. 2A) could be prominently detected only in control cells 10 min. after TNF stimulation prior to complete IκBα degradation, demonstrating that overall IκBα ubiquitination was efficiently inhibited in cells pretreated with MLN4924. IκBα‐Ub was detectable with both, a phospho‐specific IκBα antibody and an Ub‐specific antibody (Fig. 2A and B), although the latter less efficiently recognized IκBα‐Ub co‐precipitated with RelA (Fig. 2A). Collectively, these data demonstrate that (*i*) IκBα and RelA both become phosphorylated within the NF‐κB/RelA‐IκBα complex and (*ii*) that phosphorylation of one, the other, or both proteins is not sufficient by itself to mediate the dissociation of the NF‐κB/RelA‐IκBα complex.

Knowing IκBα and RelA to become simultaneously phosphorylated in the cytosol in response to TNF and their phosphorylation to occur within the NF‐κB/RelA‐IκBα complex, we next analysed the dependence of RelA phosphorylation on canonical IKKs (the IKK complex) and IKK‐related kinases (IKKε and TBK1). TNF‐induced phosphorylation and degradation of IκBα in the cytosol were completely inhibited in cells pretreated with TPCA‐1 (Fig. S2A), an inhibitor of canonical IKKs with high selectivity for IKKβ 68, demonstrating this inhibitor to efficiently block kinase activity of the IKK complex, being responsible for IκBα phosphorylation in the canonical pathway of NF‐κB activation 1, 6. Notably, phosphorylation of RelA in the cytosol at Ser468 and Ser536 was nearly completely inhibited in cells pretreated with TPCA‐1 alike (Fig. S2A), indicating TNF‐induced phosphorylation of RelA at these sites to essentially depend on IKK complex kinase activity as well, as previously suggested in some reports 63, 64. Pretreatment of cells with MRT67307, a selective inhibitor of IKK‐related kinases in contrast 69, only marginally decreased phosphorylation of IκBα and RelA in the cytosol, phosphorylation of RelA at Ser468 being most prominently affected (Fig. S2B). In contrast to TPCA‐1, but (unexpectedly) not BMS‐345541, another inhibitor of canonical IKKs reported to be selective for IKKβ 70, which delayed but did not completely prevent TNF‐induced IκBα phosphorylation/degradation and RelA phosphorylation/nuclear translocation, PI3K inhibitor wortmannin did not affect any of the cytoplasmic events (Fig. S2A). As expected, inhibition of IκBα phosphorylation and degradation by TPCA‐1 coincided with an efficient inhibition of TNF‐induced RelA nuclear translocation (Fig. S2A). In cells pretreated with MRT67307 in contrast, a slight delay in RelA nuclear accumulation, yet prolonged nuclear residence of RelA and its phosphorylated isoforms, was observed (Fig. S2B), indicating IKK‐related kinases to contribute to down‐regulation of RelA nuclear abundance. While inhibition of NF‐κB activation by IKK‐related kinases has been recently reported in murine embryonic fibroblasts stimulated with TNF, interleukin‐1 or TLR agonists 69, it remains to be explored, if inhibition of RelA nuclear residence by IKK‐related kinases in HeLa cells is because of catalytic inhibition of canonical IKKs (the IKK complex) in the cytosol 69 or a more direct impact of IKK‐related kinases on RelA (proteolytic turnover or nuclear export) within the nucleus. Our data however do not underpin a (major) contributive role of IKK‐related kinases in the phosphorylation of RelA at serines 468 and 536 in either the cytosol or the nucleus in HeLa cells, a role which was previously suggested for IKKε in other cell systems 65, 66.

### p97/VCP contributes to the maintenance of IκBα and RelA protein expression

**Figure 3:**
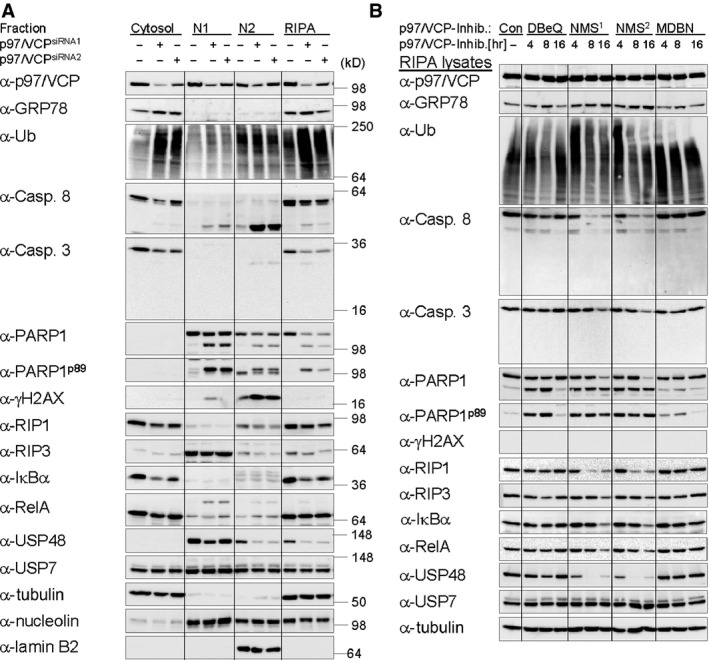
p97/VCP contributes to the maintenance of IκBα and RelA protein expression. (**A**) Knockdown of p97/VCP (72 hrs) induces apoptosis and reduces protein expression of IκBα. (**B**) Inhibition of p97/VCP with the most potent selective inhibitor NMS‐873 reduces protein expression of IκBα and RelA and induces apoptosis within hours. (**A** and **B**) Cells were harvested by subcellular fractionation or RIPA lysis, as stated, 72 hrs post RNAi of p97/VCP or at the indicated times after treatment with various p97/VCP inhibitors: DBeQ (15 μM), NMS‐873 (2.5 μM (NMS ^1^) or 5 μM (NMS ^2^)) or MDBN (15 μM). Samples were analysed by IB by use of the indicated antibodies. Tubulin (cytosol), Nucleolin (soluble nuclear fraction, N1) and Lamin B2 (insoluble nuclear fraction, N2) were used as marker proteins and/or detected for control of equal protein load. Induction of DNA damage and apoptosis was verified through IB detection of γH2AX (accumulating in N2) and/or cleavage of the nuclear protein PARP1 (detectable in N1, N2 and RIPA lysates) respectively.

The homohexameric AAA ATPase p97/VCP was previously reported to selectively associate with ubiquitinated IκBα *in vitro*, as well as in cells stimulated with TNF, and to promote its UPP‐dependent degradation in an *in vitro* assay 71. Through RNAi of p97/VCP and overexpression of either the wild‐type protein or an ATPase defective mutant, its requirement for cytokine‐induced UPP‐dependent degradation of IκBα was recently confirmed in different cell lineages. Furthermore, the UFD1L‐NPL4 heterodimer was identified as the p97/VCP adapter molecule mediating its recruitment to ubiquitinated IκBα 29. While cells depleted for p97/VCP were noted previously to express slightly diminished amounts of IκBα protein 29, we observed protein expression of several components of the NF‐κB system (IκBα, RelA, RIP1 and USP48) to be depleted in cells after separate treatment with two different p97/VCP‐specific siRNAs to different extend, which correlated with knockdown efficiency (Fig. 3A). Apart from this, depletion of p97/VCP induced accumulation of ubiquitinated proteins (predominantly in the cytosol) as well as ER stress, as expected, the latter being signified by increased expression of ER chaperone GRP78 72, 73. Furthermore, beside transient deceleration/arrest of proliferation the third day after siRNA transfection (Fig. S3), induction of apoptosis was noted, as previously reported 72, 73 and indicated by decreasing amounts of pro‐caspases 8 and 3, accumulation of cleaved caspase 8 (but not caspase 3) and accumulation of cleaved PARP1 (Fig. 3A). Interestingly, execution of apoptosis appeared to be impaired, as cells stayed attached to culture plates and recovered from growth arrest the fourth day after siRNA transfection (Fig. S3). On the basis of all these observations, we thus wondered if p97/VCP is in fact essential for cytokine‐induced proteolysis of IκBα or if accumulation of IκBα‐Ub observed in p97/VCP‐depleted cells 29 might just reflect a general deterioration of cell metabolism and proteostasis. To address this issue, we decided to subject cells to treatment with p97/VCP inhibitors prior to and during TNF stimulation, potent and selective VCP inhibitors having become available recently 38.

Long‐term effects closely resembling those observed after p97/VCP RNAi, described above, were only obtained with the allosteric p97/VCP inhibitor NMS‐873 (Fig. 3B) 73, which mediated increased expression of GRP78 as well as accumulation of ubiquitinated proteins and cleaved PARP1 within 4 hrs and a decrease in protein expression of IκBα, RelA, USP48 and RIP1 at later times. An ATP competitive (DBeQ) 74 and a covalent VCP inhibitor (MDBN) 75 were less effective. The lack of γH2AX accumulation in NMS‐873‐treated cells is because of the fact that RIPA lysis (Fig. 3B) in contrast to N2 nuclear extraction (Fig. 3A) does not solubilize chromatin‐associated proteins. Notably, the observed increase in indicators of apoptosis induction (cleavage of caspases and PARP1) did not coincide with noticeable cell loss (microscopic observation of cell rounding and detachment from culture plates) within 8 hrs of inhibitor treatment. Upon treatment overnight, however, live cells decreased. Notably, largely unimpaired cell viability within 8 hrs of NMS‐873 treatment has been reported for HCT116 colon carcinoma cells as well 73.

### p97/VCP promotes timely and efficient TNF‐induced degradation of ubiquitinated IκBα and the concomitant liberation of RelA

**Figure 4:**
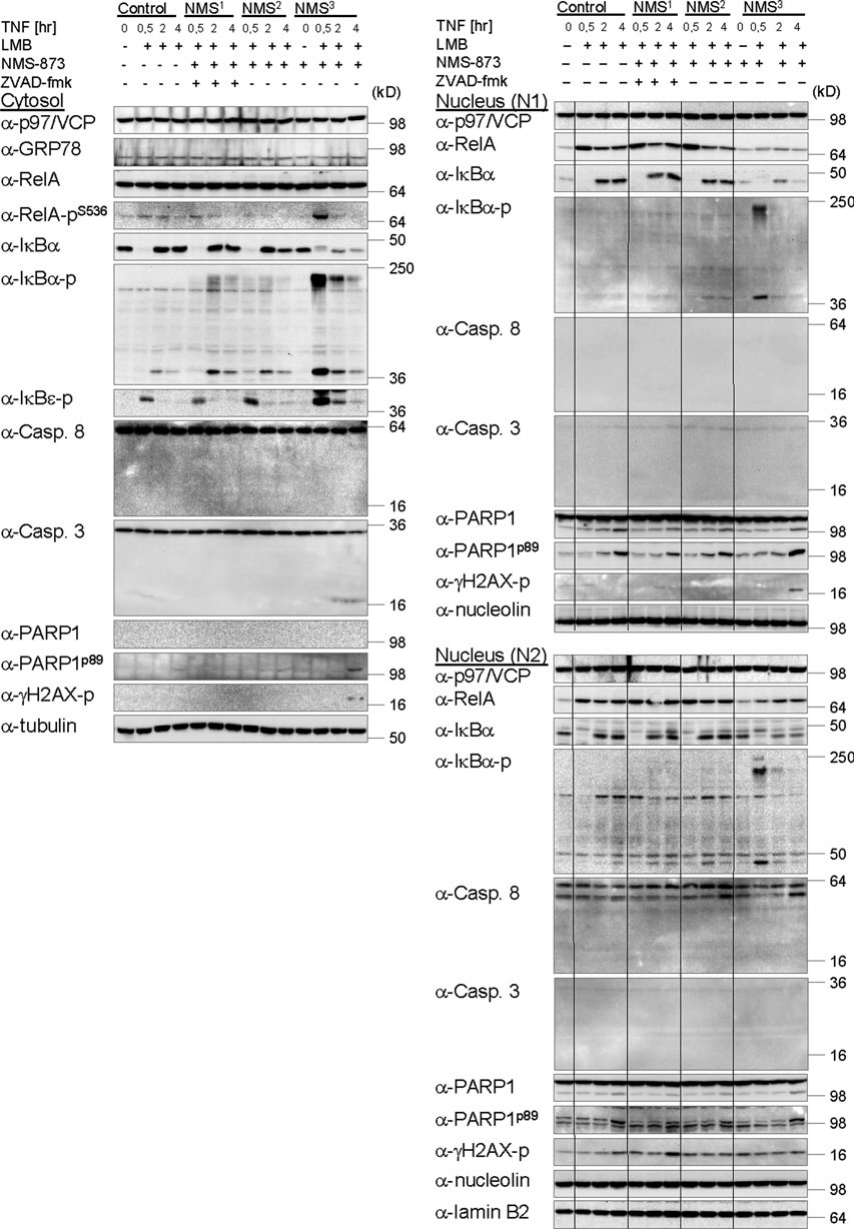
Efficient TNF‐induced degradation of IκBα and concomitant liberation of RelA requires functional p97/VCP. Cells stimulated with TNF (10 ng/ml) for the indicated times after either pretreatment with p97/VCP inhibitor NMS‐873 (2.5 μM, NMS ^3^) for 10 min. or inhibitor treatment (2.5 μM) from 15 min. after stimulation in either the presence (NMS ^1^) or the absence (NMS ^2^) of pan‐caspase inhibitor ZVAD‐fmk (10 μM), were subjected to subcellular fractionation. LMB (10 ng/ml) was applied 15 min. after stimulation to prevent Crm1‐dependent nuclear export of RelA. Samples were analysed by IB by use of indicated antibodies. Tubulin (cytosol), Nucleolin (N1) or Lamin B2 and HDAC1 (N2) were used as marker proteins for the respective subcellular fractions and detected for control of equal protein load. Detection of γH2AX on one hand and caspase and PARP1 cleavage on the other was accomplished to determine induction of DNA damage and or apoptosis respectively.

Having confirmed NMS‐873 to most potently inhibit p97/VCP 73, we next explored its impact on TNF‐induced NF‐κB activation. In cells pretreated with NMS‐873, a delay in IκBα degradation and re‐accumulation was apparent in the cytoplasm and, less prominently, in the nucleus alike (Fig. 4), which coincided with strong accumulation of phosphorylated and ubiquitinated IκBα 30 min. after TNF stimulation, where after both declined slowly. Simultaneously, phosphorylated RelA transiently accumulated only in the cytoplasm (Fig. 4). Decelerated TNF‐induced IκBα degradation in cells devoid of functional p97/VCP was accompanied by strongly impaired and delayed RelA nuclear accumulation in N1 and N2, which remained unaffected when cells were exposed to NMS‐873 15 min. after TNF stimulation, either alone, or in combination with pan‐caspase inhibitor ZVAD‐fmk, to prevent induction of apoptosis (Fig. 4). Congruently, re‐accumulation of IκBα, being a bona fide NF‐κB target gene 76, was not impaired in these cells (Fig. 4).

In cells pretreated with NMS‐873, cell viability was not significantly decreased during an 8 hrs time course of TNF stimulation compared to cells stimulated with TNF in the absence of the inhibitor (Fig. S4). In cells pretreated with cycloheximide in contrast, TNF efficiently actuated induction and execution of apoptosis within the 8 hrs time period (Fig. S4), as expected 77, 78, 79.

### Functional p97/VCP is required for timely and efficient liberation of RelA from ubiquitinated IκBα

**Figure 5:**
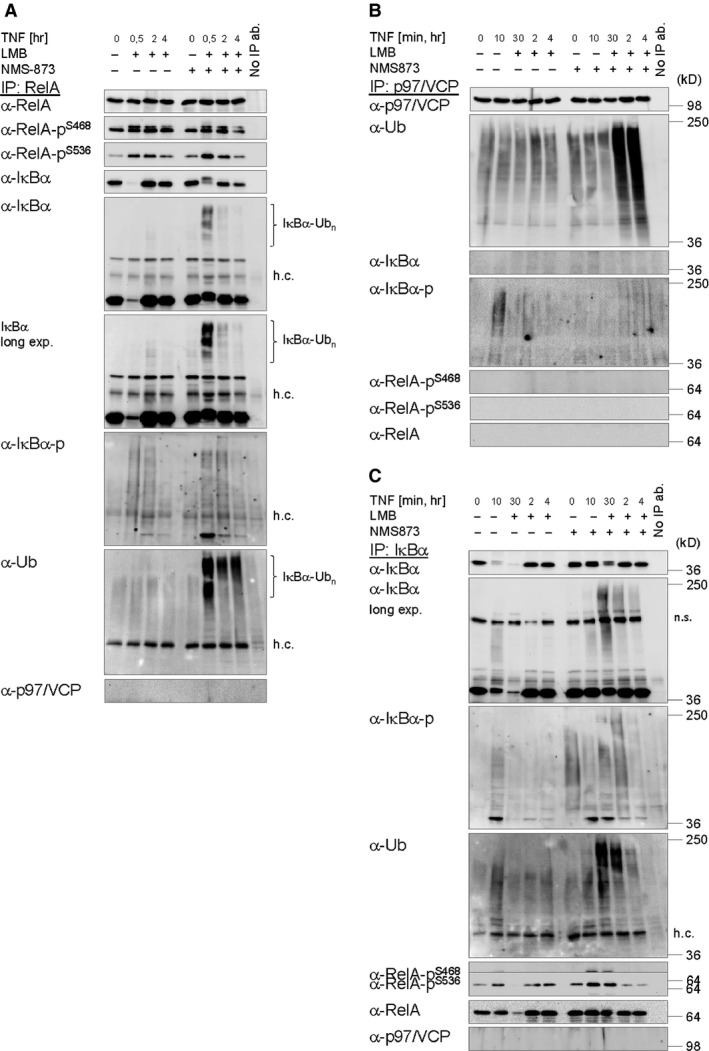
Immediate and efficient dissociation of RelA from ubiquitinated IκBα after TNF stimulation requires functional p97/VCP to subsequently allow (i) rapid and efficient proteasomal degradation of ubiquitinated IκBα and (ii) rapid and efficient RelA nuclear translocation. (**A**) Inhibition of p97/VCP promotes the co‐precipitation of ubiquitinated IκBα with RelA in response to TNF. (**B**) Inhibition of p97/VCP promotes its association with ubiquitinated proteins but not ubiquitinated IκBα in response to TNF. (**C**) Inhibition of p97/VCP prevents/decelerates the TNF‐induced liberation of RelA from ubiquitinated IκBα. (**A**–**C**) IPs of RelA (**A**), p97/VCP (**B**) or IκBα (**C**) were performed from cytosolic fractions of cells pretreated with NMS‐873 (2.5 μM) for 10 min. prior to TNF (10 ng/ml) stimulation for the indicated times. LMB (10 ng/ml) was applied 15 min. after TNF stimulation. Samples were analysed by IB using the indicated antibodies.

Analysis of RelA‐IPs performed from cytosolic fractions of cells pretreated with NMS‐873 revealed co‐precipitation of phosphorylated IκBα and IκBα‐Ub with RelA most prominently 30 min. after TNF stimulation, IκBα‐Ub being detectable with both, an IκBα‐specific and an Ub‐specific antibody, but in this case, for unknown reason, not with a phospho‐specific IκBα antibody (Fig. 5A). Like overall IκBα‐Ub in the cytoplasm, IκBα‐Ub associated with RelA slowly decreased over time. Of note, co‐precipitation of IκBα‐Ub with RelA was not detectable in control cells equipped with functional p97/VCP, consistent with it being immediately degraded *via* the UPP. p97/VCP did not co‐precipitate with RelA, as reported previously 71. Collectively, these data indicate p97/VCP to be essential for timely (rapid and efficient) cytokine‐induced degradation of IκBα *via* the UPP and a simultaneous liberation of active NF‐κB/RelA. In deviation to previous suggestions, however 29, 71, both events (although becoming strongly decelerated and inefficient) are not completely abolished in the absence of functional p97/VCP. This might suggest (activatable) bypass pathways for p97/VCP to exist in cells, which could ultimately limit an envisaged usability of p97/VCP inhibitors as anti‐cancer drugs 38, at least when applied separately.

To confirm a TNF‐induced association of p97/VCP with IκBα‐Ub 29, 71, reciprocal IPs of both interaction partners were performed. IκBα‐Ub, detectable with the phospho‐specific IκBα antibody, but not unmodified IκBα, transiently co‐precipitated with p97/VCP 10 min. after TNF stimulation in control cells but not in cells pretreated with p97/VCP inhibitor NMS‐873 (Fig. 5B). Notably, this became apparent although ubiquitinated proteins in general, associated with p97/VCP, as detected with an Ub‐specific antibody, strongly accumulated over time exclusively in NMS‐873‐treated cells. This might suggest that upon inhibitor treatment p97/VCP becomes locked in association with constitutive substrates, which, as a result, fail to be appropriately processed and liberated or delivered to the 26S proteasome. In consequence, locked p97/VCP might be prohibited from (transiently) associating with ubiquitinated IκBα in response to TNF stimulation. RelA and its phosphorylated isoforms did not co‐precipitate with p97/VCP as reported previously 71 (Fig. 5B). Unfortunately, we also failed to demonstrate a co‐precipitation of VCP with IκBα‐Ub (Fig. 5C). Analysis of IκBα‐IPs however confirmed strong accumulation of phosphorylated IκBα as well as IκBα‐Ub in cells devoid of functional p97/VCP 30 min. after TNF stimulation, both of which slowly decreased over time thereafter consistent with our previous observations (Fig. 5A). Furthermore, an enhanced association of phosphorylated RelA isoforms before and, more prominently, 10 and 30 min. after TNF stimulation was observed concomitant with a clear defect in the liberation of RelA from (ubiquitinated) IκBα 30 min. after stimulation (Fig. 5C).

### Conclusion and perspective

On the basis our data, we propose the following model: IKK complex‐dependent TNF‐induced phosphorylation of RelA at serines 468 and 536, being accomplished in the cytosol within the NF‐κB/RelA‐IκBα complex simultaneous with IKK complex‐dependent phosphorylation of IκBα, is likely catalysed by the IKK complex itself (Fig. 2 and Fig. S2A). Timely and efficient liberation of (phosphorylated) RelA from IκBα, however, additionally (indirectly but essentially) requires CRL (CRL1^β‐TrCP^)‐dependent IκBα ubiquitination, being almost completely inhibited by MLN4924 (Fig. 2A and B), and, thereafter, functional p97/VCP to promote timely and efficient separation of IκBα‐Ub from (phosphorylated) RelA through an unknown mechanism, being inhibited by NMS‐873 (Fig. 5A and C). Consequential liberation of RelA, which could additionally depend on unknown accessory proteins, might ensure RelA nuclear accumulation and p97/VCP‐promoted UPS‐dependent degradation of only IκBα‐Ub to occur concertedly (Fig. 4). In contrast to published data, however 29, p97/VCP is not absolutely required for the degradation of ubiquitinated IκBα, proceeding with noticeably slowed kinetics even in its absence (Fig. 5A and C). Under these conditions, however, IκBα‐associated RelA might be accidently co‐degraded with IκBα‐Ub to some extent, causing a loss in protein expression of IκBα and RelA (among others) over time (Fig. 3A and B). Loss of RelA protein expression might causally contribute to increased cell susceptibility for apoptosis induction (Fig. 3A and B). Yet, execution of cell death appears again to depend on functional p97/VCP (Figs S3 and S4) for reasons deserving further investigation.

Open questions additionally persist concerning the detailed mechanism of the p97/VCP‐dependent separation of IκBα‐Ub from activated RelA prior to proteolysis of only IκBα‐Ub. Apart from p97/VCP's segregase activity, relying on the generation of mechanical force through ATP hydrolysis, to extract client proteins from highly ordered structures (*e.g*. DNA or protein complexes) or membranes 80, its unfoldase/remodelling and Ub‐chain editing activities could be involved as well, both of which might facilitate capture and uptake of IκBα‐Ub by the proteasome 80, 81, 82. p97/VCP unfoldase/remodelling activity might depend or not on ATP hydrolysis 81, 83 and be driven by either the chaperone alone or in synergy with associated 26S proteasome 81. Furthermore, p97/VCP might shuttle IκBα‐Ub to either downstream Ub‐binding proteins or the proteasome 71, 82. Subsequently, or apart from this, the proteasome could be activated through mechanical coupling to p97/VCP (directly) or removal of an endogenous inhibitor [*e.g*. PSFM1 (indirectly)] by the hexameric molecule 84, 85. The fact that p97/VCP ATPase inhibitor MDBN 75 (Fig. S5) and ATP competitive p97/VCP inhibitor DBeQ 74 (Fig. S5) were less effective than allosteric inhibitor NMS‐873 73 (Fig. S5) in affecting poly‐Ub and IκBα protein expression, at least in unstimulated cells (Fig. 3B), and that NMS‐873 supposedly inhibits (potentially/presumably) p97/VCP ATPase‐independent activities (*e.g*. unfoldase 83 and Ub‐chain editing activities 35, 80 or mechanical coupling to the proteasome 84) besides (ongoing) ATP hydrolysis alike 73, suggests a combination of activities to be at play in the turnover of IκBα‐Ub. A contributive role of p97/VCP ATPase activity has, however, been demonstrated through overexpression of an ATPase‐deficient mutant 29. Regardless of their ATPase dependence, overall activities of p97/VCP, relevant for IκBα‐Ub proteolytic demise, do however appear to heavily depend on accessibility/flexibility of the linker region between the D1 and D2 ATPase domains, as well as the amenability of p97/VCP towards intramolecular motions/conformational changes (Figs 4 and 5), both of which are antagonized by NMS‐873 73. The inhibitor binding site consists of a molecular pocket lined by amino acid residues of D1 and D2 domains of adjacent protomers, which involves a lateral tunnel leading to the central pore of the hexamer, and is situated in close proximity to the D1‐D2 linker 73 (Fig. S5). Notably, it overlaps with the pore‐2 loop (aa 586‐593) located in the D2 domain, which was recently reported to be involved in mechanical coupling of the chaperone to the 20S proteasome, potentially facilitating engulfment and degradation of a subset of ubiquitinated client proteins 84. While knowledge about affected client proteins is currently lacking in humans 84, IκBα might be a candidate, IκBα‐Ub strongly accumulating in response to TNF in the presence of NMS‐873. Identification and detailed elucidation of all p97/VCP‐dependent events contributing to IκBα‐Ub degradation will indubitably require sophisticated future experimentation. Independent from ATP turnover by the p97/VCP hexamer, conformational changes within its individual subunits and motion of its subunits within the entire molecule could be induced through transient association of the chaperone with appropriate interaction partners or by posttranslational modifications of either p97/VCP itself or associated accessory proteins (*e.g*. through phosphorylation or acetylation). While several potential sites for phosphorylation and acetylation have been identified in p97/VCP 86, 87, 88, involvement of specific sites and modifying enzymes in regulation of the NF‐κB pathway and the proteolytic turnover of IκBα, in response to TNF or alternative agonists, is largely unexplored by now.

## Conflicts of interest

The authors confirm that there are no conflicts of interest.

## Supporting information


**Figure S1** In response to TNF the proteolytic turnover of p105 (NF‐κB1) and IκBε but not p100 (NF‐κB2) is regulated in a phosphorylation‐ and CRL‐dependent manner in the cytosol and the nucleus.
**Figure S2** Classical IKKs (the IKK complex) but not IKK‐related kinases (IKKε and TBK1) regulate the UPS‐dependent degradation of IκBα and the liberation of RelA in response to TNF.
**Figure S3** p97/VCP promotes cell proliferation and protects from apoptosis induction.
**Figure S4** Functional inactivation of p97/VCP does not affect cell viability upon TNF stimulation.
**Figure S5** Structural organization and sites of functional relevance in human p97/VCP.
**Table S1** Primary antibodies used in the study.
**Table S2** Secondary antibodies used in the study.
