# Supplementary material for: p97/VCP promotes Cullin‐RING‐ubiquitin‐ligase/proteasome‐dependent degradation of IκBα and the preceding liberation of RelA from ubiquitinated IκBα
Source: J Cell Mol Med. 2015 Oct 14;20(1):58–70. doi: 10.1111/jcmm.12702 (PMC4717852; doi:10.1111/jcmm.12702)

Supplemental Information

Supplemental Information encompasses 5 figures (figures S1-S5), associated figure legends and two tables (tables S1 and S2).

Supplemental Figure Legends

Figure S1 related to figure 1: In response to TNF the proteolytic turnover of p105 (NF-κB1) and IκBε but not p100 (NF-κB2) is regulated in a phosphorylation- and CRL-dependent manner in the cytosol and the nucleus. After pre-treatment with MLN4924 (3 µM) 10 min prior to TNF (10 ng/ml) stimulation, cells were harvested by subcellular fractionation at indicated times. Leptomycin B (LMB, 10 ng/ml) was added 15 min after TNF stimulation to prevent Crm1-dependent nuclear export. Samples, as indicated, were analyzed by IB. Detection of either Tubulin (cytosol) or HDAC1 and Lamin B2 (total nuclear fractions, N_t) was performed for control of fractionation success and equal protein load.

Figure S2 related to figure 2: Classical IKKs (the IKK complex) but not IKK-related kinases (IKKε and TBK1) regulate the UPS-dependent degradation of IκBα and the liberation of RelA in response to TNF. A) The IKK complex but not phosphoinositol-3-kinase (PI3K) promotes phosphorylation and UPS-dependent turnover of IκBα in response to TNF. B) IKK-related kinases are dispensable for the TNF-induced phosphorylation of IκBα and RelA as well as the UPS-dependent degradation of IκBα in the cytosol, but appear to contribute to the limitation of either the nuclear accumulation or the residence/turnover of (phosphorylated) RelA. A, B) Subcellular fractions of cells treated with either the IKKα/β-selective inhibitor TPCA-1 (10 µM), the IKKα/β-selective inhibitor BMS-345541 (25 µM), the PI3K inhibitor wortmannin (10 µM) or the selective IKKε/TBK1 inhibitor MRT67307 (10 µM), as indicated, 20 min prior to TNF stimulation (10 ng/ml) were prepared at indicated times. Cells were not treated with Leptomycin B. Samples, as indicated, were analyzed by IB. Detection of Tubulin (cytosol), Nucleolin (soluble nuclear fraction, N1) or HDAC1 and Lamin B2 (insoluble nuclear fraction, N2) was performed for control of fractionation success and equal protein load.

Figure S3 related to figure 3: p97/VCP promotes cell proliferation and protects from apoptosis induction. RNAi-mediated depletion of p97/VCP (transiently) decelerates cell proliferation and initiates apoptosis. Cells (6.000/well) were seeded in two triplicates 1 day after siRNA treatment. Every 24h cell numbers (mean, n = 6, left panel) were determined by use of Cell Titer-Glo® Luminescent Cell Viability Assay. p97/VCP knockdown efficiency and induction of apoptosis were analyzed by IB analysis of RIPA cell lysates the third day after siRNA transfection (insert). Differences between control and knockdown cells at selected times after siRNA transfection are additionally presented in a bar chart diagram (mean cell numbers +/- s.d., n = 6, right panel). All data derive from one representative experiment. (*, p ≤ 0,01).

Figure S4 related to figure 4: Functional inactivation of p97/VCP does not affect cell viability upon TNF stimulation. Cells (20.000/well) were seeded in triplicate one day prior to serum starvation overnight. Serum-starved cells were then exposed to either p97/VCP inhibitor NMS-873 (2,5 µM, 20 min) or CHX (30 µg/ml, 5 min) prior to TNF stimulation (10 ng/ml in perpetuated presence of the inhibitors) for the indicated times. Afterwards, Cell Titer-Glo® Luminescent Cell Viability Assay, calibrated to determine cell numbers, was performed. Data (mean cells numbers [%] +/- s.d. [CV%]; n = 3) obtained from one representative experiment are presented. (*, p ≤ 0,01).

Figure S5 related to figures 3-5: Structural organization and sites of functional relevance in human p97/VCP. The structural organization of human p97/VCP (P55072.4) is illustrated.

Borders of domains and motifs, as well as well as the location of relevant functional sites were retrieved from the NCBI Protein Database. N1 and N2, subdomains of the p97/VCP N-domain; D1 and D2, p97/VCP ATPase domains; D1α and D2α, C-terminal α-helical subdomains of the ATPase domains; C, C-terminal oligomerization domain; WA and WB, Walker A (aa 245-252 and aa 518-525) and Walker B motifs (aa 300-305 and aa 573-578) of the ATPase domains, involved in nucleotide binding (WA) and ATP hydrolysis (WB) respectively [Wang et al., 2005]; R-finger, arginine finger, being part of the second region of homology (SRH), situated in the ATPase domains, which is involved in interprotomer communication and wiring of conformational changes within the p97/VCP hexamer during ATP hydrolysis [Wang et al., 2005]. The SRH of the D1 domain additionally contributes to poly-Ub-substrate binding of the N-domain and cofactor communications with the chaperone [Wang et al., 2005]. Under physiologic conditions, the D2 domain is responsible for the majority of p97/VCP ATPase activity, whereas (stable) nucleotide binding in the D1 domain [DeLaBarre and Brunger, 2003] primarily contributes to hexamer formation and binding of ubiquitinated client proteins with the help of adapter proteins [Wang et al., 2005], e.g. the UFD1L-NPL4 heterodimer involved in the association of p97/VCP with IκBα-Ub [Li et al., 2014]. The Following information is additionally provided: (I) Binding sites for p97/VCP adapter proteins, being recruited to the p97/VCP N-domain or C-terminus via the indicated domains or motifs present in the adapter proteins [Meyer and Weihl, 2014]. Association of the UFD1L-NPL4 heterodimer with the p97/VCP N-domain is accomplished via a UBX-L domain and a SHP motif located in NPL4 and UFD1L respectively [Meyer and Weihl, 2014]. (II) The epitopes recognized by different p97/VCP-specific antibodies available from *Santa Cruz Biotechnology* (sc-catalogue number) and *Abcam* respectively (ab catalogue number). The antibodies from *Abcam* are functional according to our experience, but were not applied in the present study. (III) The target sites of p97/VCP inhibitors, including their mechanism of action, according to published reports [Chou et al., 2011; Chou and Deshaies, 2011; Magnaghi et al., 2013]. NMS-859 [Magnaghi et al., 2013] shares the mechanism of action with MDBN, but was not used in the present study. The binding site for allosteric p97/VCP inhibitor NMS-873 was determined [Magnaghi et al., 2013].

Supplemental References

Chou, T.-F., Brown, S.J., Minond, D., Nordin, B.E., Li, K., Jones, A.C., Chase, P., Porubsky, P.R., Stoltz, B.M., Schoenen, F.J., Patricelli, M.P., Hodder, P., Rosen, H. and Deshaies, R.J. **(2011)**. Reversible inhibitor of p97, DBeQ, impairs both ubiquitin-dependent and autophagic protein clearance pathways. *Proc. Natl. Acad. Sci. U.S.A.* **108:** 4834-4839

Chou, T.-F., and Deshaies, R.J. **(2011)**. Quantitative cell-based protein degradation assays to identify and classify drugs that target the ubiquitin-proteasome system. *J. Biol. Chem.* **286:** 16546-16554

DeLaBarre, B., and Brunger, A.T. **(2003)**. Complete structure of p97/valosin-containing protein reveals communication between nucleotide domains. *Nature Struct. Biol.* **10:** 856-863

Li, J.-M., Wu, H., Zhang, W., Blackburn, M.R. and Jin, J. **(2014)**. The p97-UFD1L-NPL4 protein complex mediates cytokine-induced IκBα proteolysis. *Mol. Cell. Biol.* **34:** 335-347

Magnaghi, P., D'Alessio, R., Valsasina, B., Avanzi, N., Rizzi, S., Asa, D., Gasparri, F., Cozzi, L., Cucchi, U., Orrenius, C., Polucci, P., Ballinari, D., Perrera, C., Leone, A., Cervi, G., Casale, E., Xiao, Y., Wong, C., Anderson, D.J., Galvani, A., Donati, D., O'Brien, T., Jackson, P.K. and Isacchi, A. **(2013)**. Covalent and allosteric inhibitors of the ATPase VCP/p97 induce cancer cell death. *Nature Chem. Biol.* **9:** 548-556

Meyer, H., and Weihl, C.C. **(2014)**. The VCP/p97 system at a glance: connecting cellular function to disease pathogenesis. *J. Cell Sci.* **127:** 1-7

Wang, Q., Song, C., Irizarry, L., Dai, R., Zhang, X., and Li, C.-C.H. **(2005)**. Multifunctional roles of the conserved Arg residues in the conserved region of homology of p97/Valosin-containing protein. *J.Biol. Chem.* **280:** 40515-40523

Supplemental Tables

Table S1: Primary antibodies used in the study

Antibody Target	Host Species	Supplier	Catalogue Number
Caspase 3	rabbit	Cell Signaling Technology	#9662
Caspase 8 (1C12)	mouse	Cell Signaling Technology	#9746
Cullin 1 (AS97)	mouse	Santa Cruz Biotechnology	sc-12761
Cullin 1	rabbit	Abcam	ab2964
Cullin 2 (C4)	mouse	Santa Cruz Biotechnology	sc-166506
Cullin 2	rabbit	Zytomed	203-1313
Cullin 3	mouse	BD Biosciences	611848
Cullin 4A/B	rabbit	Epitomics	#2527-1
Cullin 5	rabbit	Bethyl Laboratories	A302-173A
Cyclin E	mouse	BD Biosciences	554192
GRP78 (H-129)	rabbit	Santa Cruz Biotechnology	sc-13968
H2AX-p^{S139} (γH2AX)	mouse	Abcam	ab26350
HDAC1 (H-51)	rabbit	Santa Cruz Biotechnology	sc-7872
IκBα (C21)	rabbit	Santa Cruz Biotechnology	sc-371
IκBα-p$^{S32/S36}$ (5A5)	mouse	Cell Signaling Technology	#9246
IκBε-p$^{S18/S22}$	rabbit	Cell Signaling Technology	#4924
Lamin B2 (O-22)	rabbit	Santa Cruz Biotechnology	sc-133722
NEDD8	rabbit	Invitrogen	34-1400
Nucleolin/C23 (H-250)	rabbit	Santa Cruz Biotechnology	sc-13057
PARP1	mouse	BD Biosciences	551025
PARP1^{p89} (cleaved PARP1)	mouse	Cell Signaling Technology	#9548
p50/p105 (H-119)	rabbit	Santa Cruz Biotechnology	sc-7178
p52/p100	rabbit	Upstate/Millipore	#05-361
p100-p$^{S864/S868}$ (NF-κB2-p)	rabbit	Cell Signaling Technology	#8410
p105-p^{S933} (NF-κB1-p)	rabbit	Cell Signaling Technology	#4806
p53 (DO-1)	mouse	Santa Cruz Biotechnology	sc-126
RelA (C20)	rabbit	Santa Cruz Biotechnology	sc-372
RelA	mouse	BD Biosciences	610869
RelA-p^{S468}	rabbit	Cell Signaling Technology	#3039
RelA-p^{S536}	rabbit	Cell Signaling Technology	#3031
RIP1	mouse	BD Biosciences	610458
RIP3	rabbit	Abcam	ab56164
Tubulin (β-Tubulin)	mouse	Sigma Aldrich	T4026
Ubiquitin (FK2)	mouse	ENZO Life Sciences	BML-PW8810
USP7	rabbit	Bethyl Laboratories	A300-033A
USP48	rabbit	Abcam	ab72226
VCP/p97 (H-120)	rabbit	Santa Cruz Biotechnology	sc-20799 (IB)
VCP/p97 (5)	mouse	Santa Cruz Biotechnology	sc-57492 (IP)

Table S2: Secondary antibodies used in the study

Antibody Target	Host Species	Supplier	Catalogue Number
anti-mouse IgG HRP (H+L)	donkey	Dianova	715-035-151
anti-mouse IgG HRP (light chain-specific)	goat	Dianova	115-035-174
anti-rabbit IgG HRP (H+L)	donkey	Dianova	711-036-152
anti-rabbit IgG HRP (light chain-specific)	mouse	Dianova	211-032-171

Fig S1 Neddylation is required in TNF-induced regulation of p105, but not p100

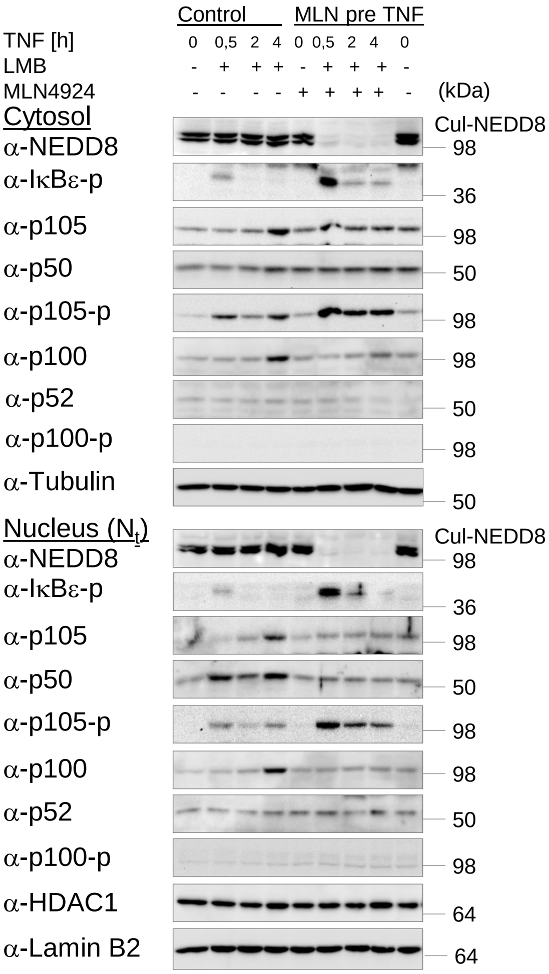

Fig S2 IKK complex, but not IkKε mediates the degradation of IκBα and the release of RelA

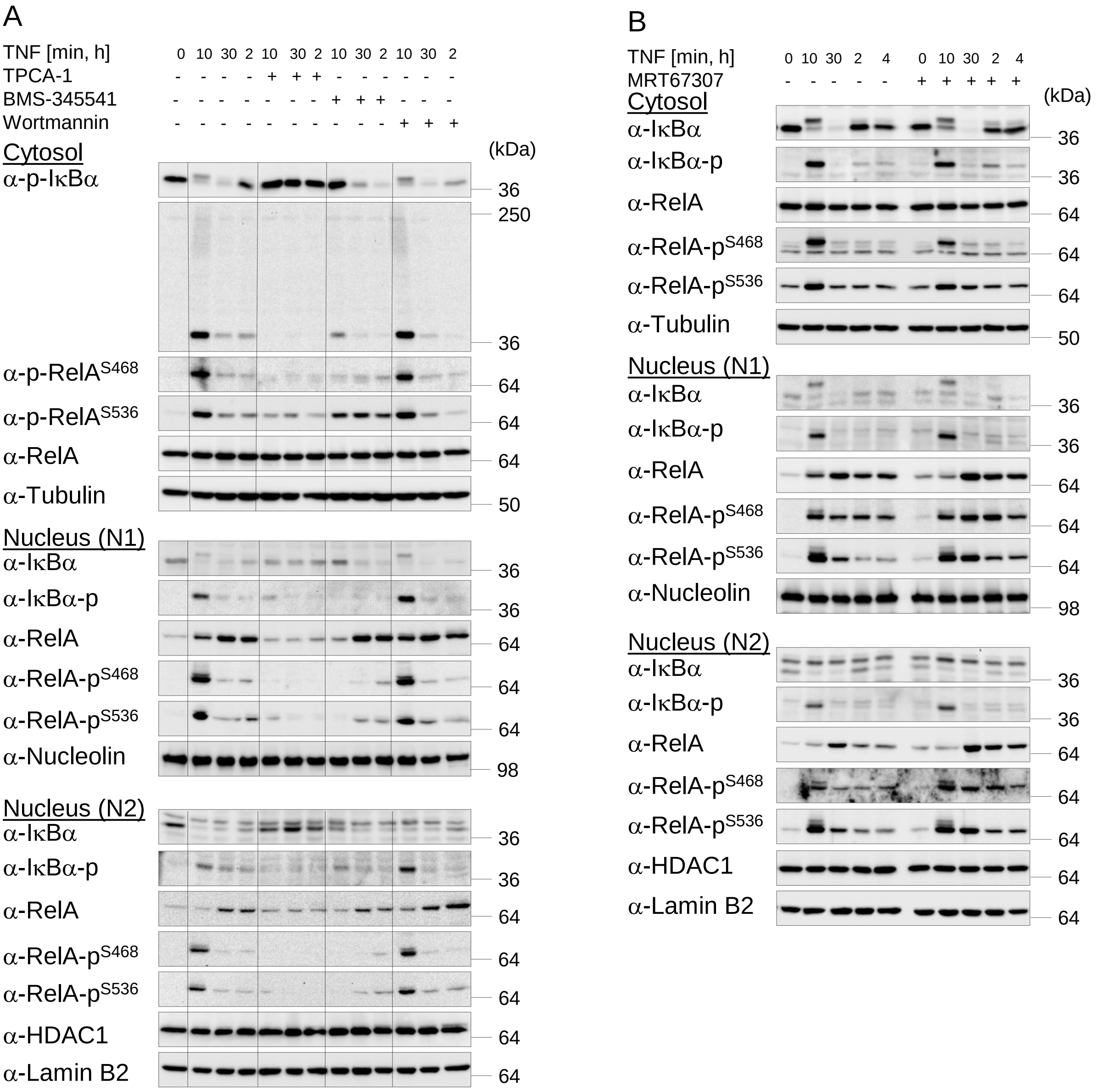

Fig S3 p97/VCP promotes cell proliferation and protects from apoptosis induction

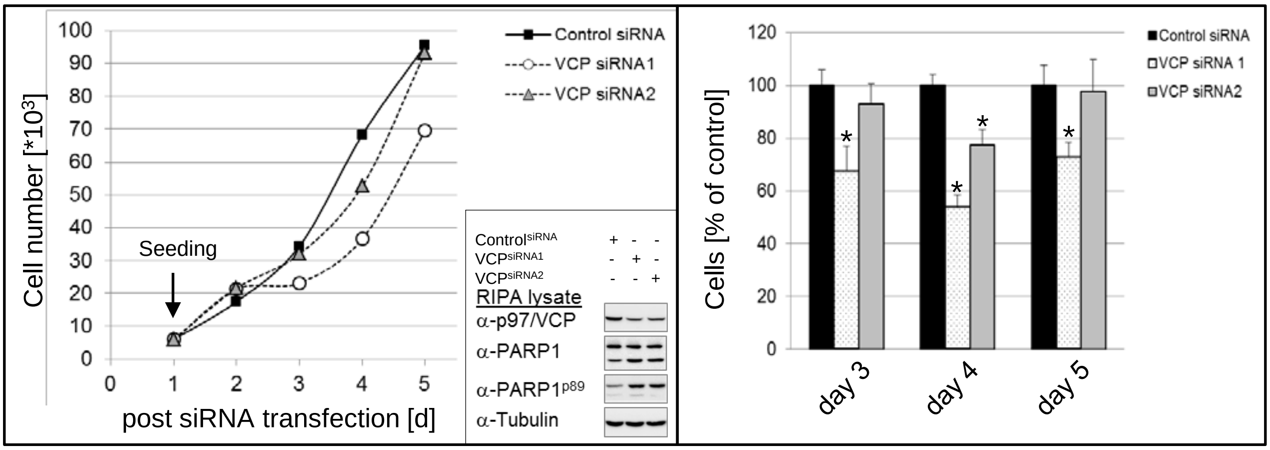

Fig S4 Functional inactivation of p97/VCP does not affect cell viability upon TNF stimulation

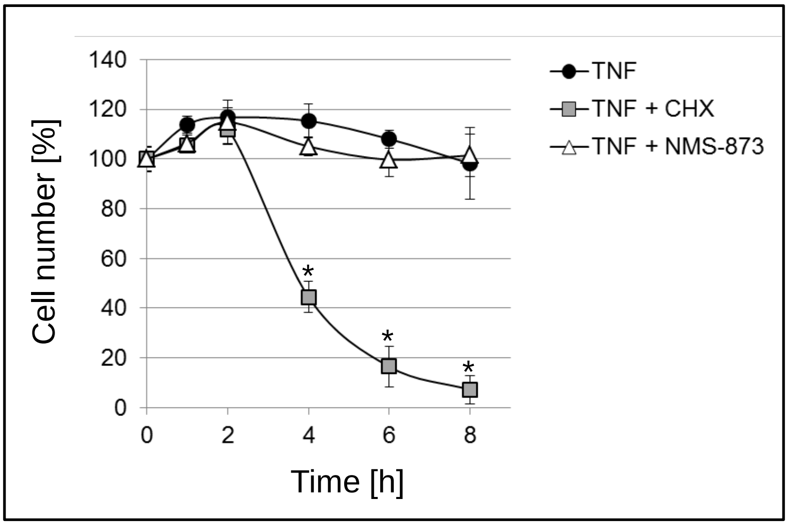

Fig S5 Structural organization and sites of functional relevance of human p97/VCP

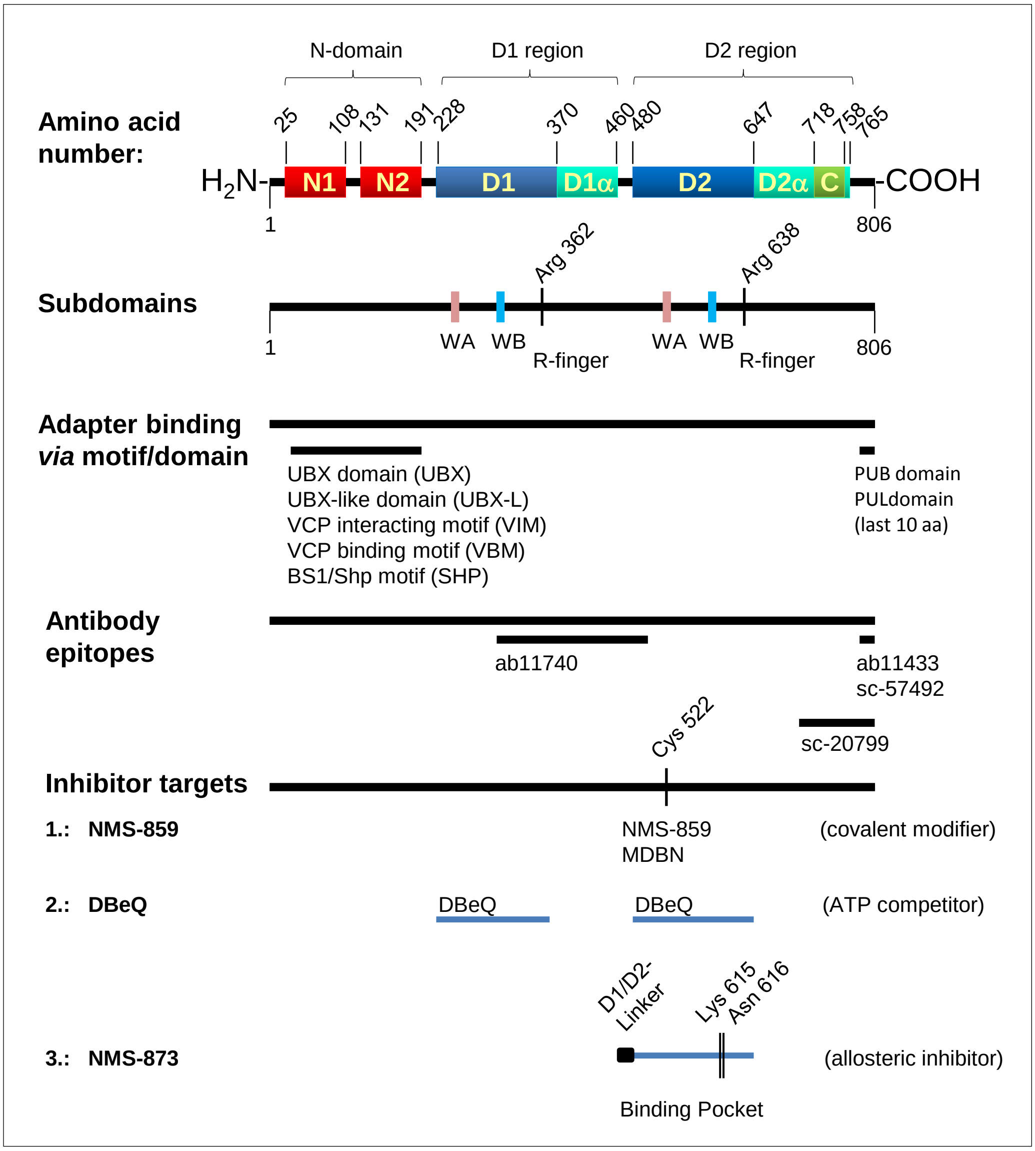

Supplement: Supplementary file 1 — Figure S1 In response to TNF the proteolytic turnover of p105 (NF‐κB1) and IκBε but not p100 (NF‐κB2) is regulated in a phosphorylation‐ and CRL‐dependent manner in the cytosol and the nucleus. Figure S2 Classical IKKs (the IKK complex) but not IKK‐related kinases (IKKε and TBK1) regulate the UPS‐dependent degradation of IκBα and the liberation of RelA in response to TNF. Figure S3 p97/VCP promotes cell proliferation and protects from apoptosis induction. Figure S4 Functional inactivation of p97/VCP does not affect cell viability upon TNF stimulation. Figure S5 Structural organization and sites of functional relevance in human p97/VCP. Table S1 Primary antibodies used in the study. Table S2 Secondary antibodies used in the study. [file JCMM-20-058-s001.pdf]
